# Comparison of 11 respiratory pathogens among hospitalized children before and during the COVID-19 epidemic in Shenzhen, China

**DOI:** 10.1186/s12985-021-01669-y

**Published:** 2021-10-09

**Authors:** Li Li, Heping Wang, Ailiang Liu, Rongjun Wang, Tingting Zhi, Yuejie Zheng, Yanming Bao, Yunsheng Chen, Wenjian Wang

**Affiliations:** 1 Department of Respiratory Diseases, Shenzhen Children’s Hospital, No. 7019 Yitian Road, Futian District, Shenzhen, 518038 Guangdong China; 2 Department of Microbiology and Immunology, College of Basic Medicine, Guangdong Medical University, Dongguan, 523808 China

**Keywords:** SARS-CoV-2, Respiratory pathogens, Prevalence, Children

## Abstract

**Background:**

The effect of SARS-CoV-2 on existing respiratory pathogens in circulation remains uncertain. This study aimed to assess the impact of SARS-CoV-2 on the prevalence of respiratory pathogens among hospitalized children.

**Methods:**

This study enrolled hospitalized children with acute respiratory infections in Shenzhen Children’s Hospital from September to December 2019 (before the COVID-19 epidemic) and those from September to December 2020 (during the COVID-19 epidemic). Nasopharyngeal swabs were collected, and respiratory pathogens were detected using multiplex PCR. The absolute case number and detection rates of 11 pathogens were collected and analyzed.

**Results:**

A total of 5696 children with respiratory tract infection received multiplex PCR examination for respiratory pathogens: 2298 from September to December 2019 and 3398 from September to December 2020. At least one pathogen was detected in 1850 (80.5%) patients in 2019, and in 2380 (70.0%) patients in 2020; the detection rate in 2020 was significantly lower than that in 2019.The *Influenza A* (InfA) detection rate was 5.6% in 2019, but 0% in 2020. The detection rates of *Mycoplasma pneumoniae*, *Human adenovirus*, and *Human rhinovirus* also decreased from 20% (460), 8.9% (206), and 41.8% (961) in 2019 to 1.0% (37), 2.1% (77), and 25.6% (873) in 2020, respectively. In contrast, the detection rates of *Human respiratory syncytial virus*, *Human parainfluenza* virus, and *Human metapneumovirus* increased from 6.6% (153), 9.9% (229), and 0.5% (12) in 2019 to 25.6% (873), 15.5% (530), and 7.2% (247) in 2020, respectively (*p* < 0.0001).

**Conclusions:**

Successful containment of seasonal influenza as a result of COVID-19 control measures will ensure we are better equipped to deal with future outbreaks of both influenza and COVID-19.Caused by virus competition, the detection rates of *Human respiratory syncytial virus*, *Human parainfluenza* virus, and *Human metapneumovirus* increased in Shenzhen,that reminds us we need to take further monitoring and preventive measures in the next epidemic season.

## Background

Coronavirus disease 2019 (COVID-19), which is caused by severe acute respiratory syndrome coronavirus 2 (SARS-CoV-2), has caused a substantial health burden worldwide. A massive roll out of population-level nonpharmaceutical interventions have been conducted to curb transmission, including stay-at-home orders, the closure of schools and retail spaces, mandated face masks in public, and encouragement of social distancing and hand hygiene. Studies from France, Japan, Singapore, Mexico, and Zhejiang province of China have shown that these measures also coincided with a decline in the number of cases of influenza over the same time period, as compared with previous seasons [1–5]. However, few studies of how seasonal other respiratory pathogens changed during the epidemic. To assess changes in seasonal respiratory viruses during the outbreak of COVID-19 in Shenzhen, hospitalized children with respiratory infections were recruited in Shenzhen Children’s Hospital. We compared the absolute case number and detection rates of 11 pathogens in the period from September to December 2019 with those from September to December 2020. Shenzhen is a large migratory city located in southern China. Since the outbreak of the epidemic, Shenzhen has been in the normalization of epidemic prevention and control, From September to December 2020, all the schools and kindergartens were opened, but the wearing of masks, social distancing, and avoidance of gathering-related activities continued to be required. On the way to and from school, students had to wear masks, there was no need to wear masks in school. Children under 2 years of age did not need to wear masks. There were strict boarder controls during this time in Shenzhen. Anyone entering via the Shenzhen port were transferred directly from the port to designated hotels for isolation for at least 14 days and may be transferred to designated hospitals for further treatment if SARS-CoV-19 PCR was tested positive during this time. During the study period, the number of confirmed COVID-19 cases in Shenzhen raised from 465 cases on 1st August 2020 to 482 cases on 31st December 2020 (17 new cases) and all were imported cases.

## Methods

### Patients

Patients with acute respiratory infections (ARIs) between September to December 2019 and September to December 2020 admitted to the pediatric wards were enrolled in Shenzhen Children’s Hospital. The inclusion criteria were as follows: age below 14 years and one or more respiratory symptoms (cough, sore throat, body temperature above 37.5 °C, and dyspnea/tachypnoea). The study protocol was approved by the Ethical Committee of Shenzhen Children’s Hospital (number: 201601304). Written informed consent was obtained from the participants’ guardians. Clinical and demographic data, as well as samples for testing pathogens, were collected by trained nurses in line with a standardized protocol.

### Specimens and detection of pathogens

Nasopharyngeal swabs were obtained by trained personnel following standard operating procedures within 24 h after admission. The specimens were transported immediately to the laboratory in sterile viral transport media. The total nucleic acids of each specimen were extracted using EasyPure Viral DNA/RNA Kit (TransGen Biotech, Beijing, China) in accordance with the manufacturer's instructions. Eleven common respiratory pathogens, including *Influenza A* (InfA), *Influenza B* (InfB), *Human parainfluenza virus* (HPIV), *Human respiratory syncytial virus* (RSV), adenoviruses (AdV), *Human metapneumovirus* (HMPV), *Human rhinovirus* (HRV), *Human bocavirus* (HBoV), *Human coronavirus* (HCoV), *Chlamydia* (Ch), and *Mycoplasma pneumoniae* (*MP*), were detected using a GeXP-based multiplex reverse transcription polymerase chain reaction (PCR) assay (Ningbo).

### Statistical analysis

Statistical analyses were conducted using SPSS 23 (SPSS Inc. Chicago, IL, USA). For comparison of categorical data, chi-square or Fisher’s exact test was used. *P*  < 0.05 was considered statistically significant.

## Results

### Patient characteristics

A total of 5696 children with respiratory tract infection were enrolled in this study, 2298 in the period from September to December 2019 and 3398 in the period from September to December 2020. The age ranged from 1 month to 14 years, and the median age was 27.5 months in 2019 and 24 months in 2020. We divided the patients according to age into four groups, as follows: (1) infant group (1 month–1 year old), 713 cases in 2019 and 1071 cases in 2020; (2) toddler group (1–3 years old), 641 cases in 2019 and 1036 cases in 2020; (3) pre-school group (3–6 years old), 647 cases in 2019 and 932 cases in 2020; (4) school children group (6–14 years old), 297 cases in 2019 and 359 cases in 2020. The subjects included 1389 boys (60.5%) and 909 girls (39.5%) (sex ratio, 1.53:1) in 2019, and 2023 boys (59.5%) and 1375 girls (40.5%) (sex ratio, 1.48:1) in 2020. There was no significant difference in sex ratio between 2019 and 2020 (X^2^ = 0.4714, *P* = 0.4913).

### Overall detection percentage of the 11 pathogens

In 2019, of 2298 specimens, 1850 (80.5%) tested positive for at least one of the 11 pathogens; in 1295 (70.0%) of these positive patients, single pathogen was detected, whereas in 555 (30.0%) patients, more than two pathogens were detected. In 2020, of 3398 specimens, 2380 (70.0%) tested positive for at least one of the 11 pathogens; in 2034 (85.5%) of these positive specimens, single pathogen was detected, and in 346 (14.5%) of patients, there were more than two pathogens. The detection rate in 2020 was significantly lower than that in 2019 (*X*^*2*^ = 78.5290, *P* = 0.000).

### Changes of specific pathogens between 2019 and 2020

The top three pathogens were HRV, MP, and HPIV in 2019, whereas HRV, RSV, and HPIV were dominant in 2020. Five pathogens (InfA, HRV, MP, AdV, and Ch) had a lower detection rate in 2020 than in 2019. The detection rate of InfA was 5.6% in 2019, but none in 2020. In contrast, the detection rates of RSV, HPIV, and HMPV in 2020 increased compared with those in 2019, where the detection rate of RSV showed the most significant increase, from 6.6 to 20.1%. Other pathogens, such as HBoV, InfB, and HCoV, showed similar detection rates between 2019 and 2020 (Table 1).

**Table 1:** Comparison of positive rates of 11 respiratory pathogens in 2019 and 2020, the detection rate of InfA was 5.6% in 2019, but none in 2020

Pathogens	2019 N = 2298	2020 N = 3398	*X*^2^	*P*
Human rhinovirus (HRV)	961 (41.8%)	873 (25.6%)	163.3277	0.000
Mycoplasma pneumoniae (MP)	460 (20%)	37 (1.0%)	616.7384	0.000
Human parainfluenza virus (HPIV)	229 (9.9%)	530 (15.5%)	37.6529	0.000
Human adenovirus (AdV)	206 (8.9%)	77 (2.2%)	130.2703	0.000
Human respiratory syncytial virus (RSV)	152 (6.6%)	683 (20.1%)	199.2841	0.000
Influenza A virus (InfA)	130 (5.6%)	0	196.7177	0.000
Human bocavirus (HBoV)	87 (3.7%)	152 (4.4%)	1.6111	0.2040
Human coronavirus (HCoV)	66 (2.8%)	123 (3.6%)	2.3891	0.1222
Chlamydia (Ch)	29 (1.2%)	22 (0.6%)	5.8343	0.0157
Human metapneumovirus (HMPV)	12 (0.5%)	247 (7.2%)	143.7734	0.000
Influenza B virus (InfB)	3 (0.1%)	1 (< 0.1%)	0.8164	0.3662

In contrast, the detection rates of RSV, HPIV, and HMPV in 2020 increased compared with those in 2019, other pathogens, such as HBoV, InfB, and HCoV, showed similar detection rates between 2019 and 2020

### Changes in specific pathogens based on month and age

**Fig. 1:**
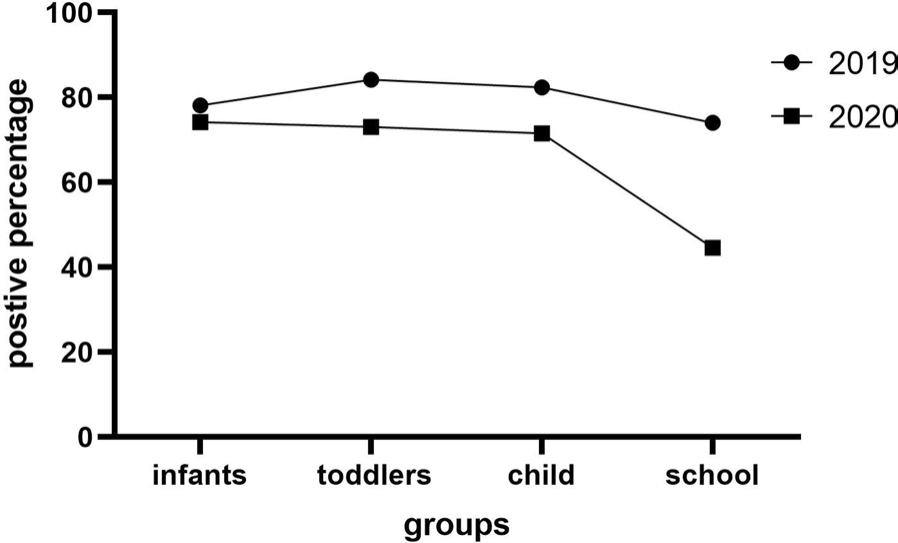
The detection rate of pathogens in four age groups in 2019 and 2020. The detection rate in school children in 2020 was lower than that in 2019, but no differences between the two years were found in other age groups

**Fig. 2:**
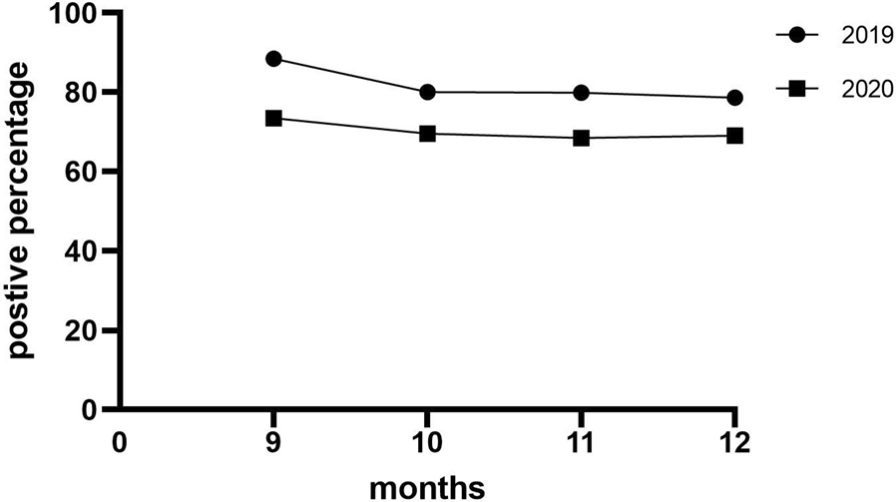
The detection rate of pathogens during four months in 2019 and 2020. The detection rate decreased from September to December in both years

The detection rate of pathogens in school children in 2020 was lower than that in 2019, but no significant differences between the two years were found in other age groups (Fig. 1). There was no difference in monthly trend of the detection rate between 2019 and 2020; in both years, the detection rate decreased from September to December (Fig. 2).

**Fig. 3:**
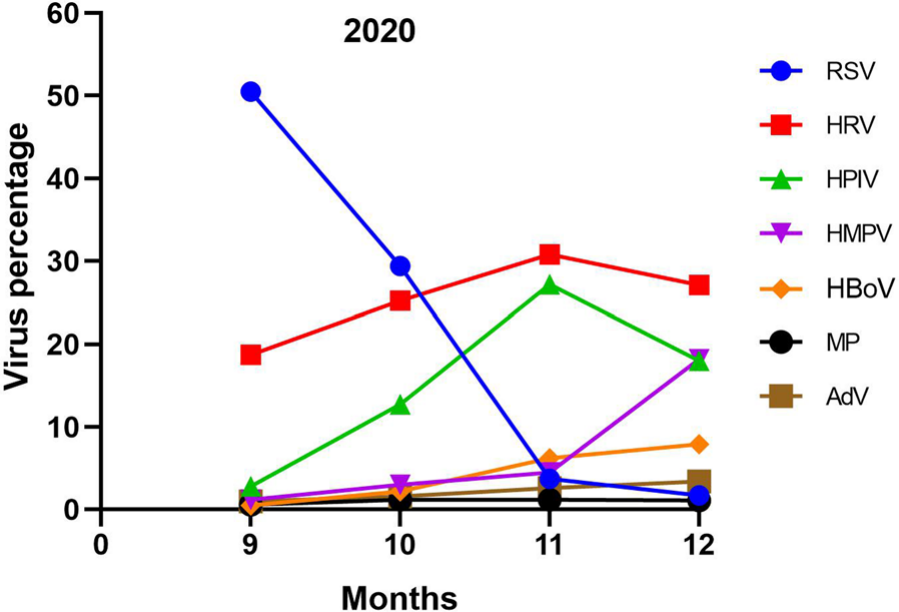
The detection rate of RSV, HRV, HPIV, HMPV, HBoV, MP, and AdV during four months in 2020

We also found some epidemic patterns of the viral infections from September to December, especially in 2020 after the epidemic. RSV was the most prevalent pathogen between September and October, and its occurrence sharply declined in November. In contrast, HPIV increased significantly in November 2020, while HMPV increased significantly in December 2020 (Fig. 3).

## Discussion

In this study, we retrospectively evaluated the overall prevalence of the most frequent respiratory pathogens among hospitalized children with ARIs before and during COVID-19 epidemic. The total positive rates of pathogens were 80.5% in 2019 and 70% in 2020, similar to those reported in other studies (from 62.4 to 85.2%) [6–8]. More specimens were tested in 2020 than in 2019, but the overall pathogens positive rate was significantly lower.

The detection rates of InfA, *MP*, HRV, and AdV decreased significantly, which led to a reduced use of oseltamivir for chemoprophylaxis and empirical use of macrolides, albeit in the epidemic season.

Droplet and contract transmission are believed to be the major transmission route of COVID-19 [9, 10]; influenza has the similar route of transmission like COVID-19. Thus, many studies have shown that nonpharmaceutical interventions associated with reduced transmission of COVID-19 have also likely substantially reduced influenza [11, 12]. Indeed, the 2019–2020 influenza season was marked by decreased influenza transmission during the COVID-19 pandemic [13]. Shenzhen is located in southern China; it has a typical subtropical monsoon climate, where influenza is mostly detected in autumn and winter [14]. Despite the school year, resumed work, and the seasonal prevalence, the influenza detection rate remained low in 2020. The potential impact of the COVID-19 pandemic on influenza is not fully known, but the reason for the observed decrease may be multifactorial. First, the COVID-19 pandemic has altered health seeking behaviors and has increased attention to nonpharmaceutical interventions that reduce the risk of transmission of influenza [13]. Other factors, such as virus competition, may have contributed as well. Namely, Lubna Pinky showed that blocking one virus infection by the presence of another can be explained simply through resource competition [15]. It has been suggested that other mechanisms, such as the immune response or interference through viral proteins, are also responsible for the growth interference between two viruses [16]. Successful containment of seasonal influenza as a result of COVID-19 control measures has provided some useful insights into controlling emerging influenza epidemics in the future.

We found that several pathogens, including RSV, HPIV, and HMPV, showed increased detection rates during the epidemic. RSV showed the most prominent increase, from 6.6 to 20.1%, what was different from other studies [17, 18], Although there was an increased detection rate of RSV, these patients did not require specific antiviral treatment in most cases. However, isolation of RSV patients was recommended to prevent spreading of disease. RSV is detected primarily during the spring and summer in Shenzhen and is the most prevalent virus in infants [14]; in our study, the detection rate of RSV gradually decreased from September to December in both years (Fig. 2). RSV disease occurs across all ages, but it disproportionately affects children under the age of 2 years [19]; since wearing mask is difficult for children under 2 years of age, an epidemiological interference between RSV and influenza virus infections has been reported: the RSV epidemic is interrupted by an epidemic of influenza virus infection [20]. According to Takeuchi’s report, the admission number of patients with RSV infection decreased during the influenza epidemic period, suggesting the presence of an epidemiological interference between RSV and influenza virus infections [21]. No cases of InfA virus were detected in 2020, which might be the reason why RSV increased in 2020. In the 2019 and 2020 two years, we found that the detection rate of RSV decreased gradually from September to November, while the detection rate of HPIV increased gradually, and both decreased in December. It may be due to a phenomenon called viral interference where one virus blocks the growth of another virus [22]. In December 2020, the detection rate of HMPV increased significantly, while the detection rate of most pathogens decreased, and it seems that COVID-19 has no impact on the prevalence of HMPV. Mandy Jongbloed et al. has showed that HMPV and SARS-CoV-2 are probably co-circulating independently [23]. Continued monitoring of viral epidemic trends and further research are needed.

This study is limited in that it was performed in a single center in a restricted geographical area, in a short period instead of a whole year or longer period, and there was a preselection bias stemming from the fact that all of the patients were hospitalized. Moreover, we did not observe changes in bacteria that cause respiratory infections, which limits our observation range; We will continue our observation during the rest of the COVID-19 outbreak in a perspective, multi-centered, and larger sample sized study to provide more precise information of the changes of the pathogenic patterns in children from South China region. This could provide some useful insights for future control of emerging respiratory pathogens epidemics.

## Conclusions

Nonpharmaceutical interventions used to prevent the spread of SARS-CoV-2 is also effective to reduce the transmission of influenza, especially during the early stage of a pandemic when there is a shortage of antiviral drugs and unavailability of specific vaccines. These information are important for us to plan strategies to prevent future influenza pandemic. However, there is an increased RSV rates as a result of the reduction of influenza prevalence. Thus, there is a need for close monitoring of the occurrence of RSV in the general population in the next RSV pandemic season. Further studies are needed to better understand the entire impact of COVID-19 on other pathogens.

## Data Availability

The key information and data generated and/or analyzed during this study were included in this article.

## Abbreviations

InfA: Influenza A
InfB: Influenza B
HPIV: Human parainfluenza virus
RSV: Human respiratory syncytial virus
AdV: Adenoviruses
HMPV: Human metapneumovirus
HRV: Human rhinovirus
HBoV: Human bocavirus
HCoV: Human coronavirus
Ch: Chlamydia
MP: Mycoplasma pneumoniae
PCR: Polymerase chain reaction
